# Knowledge mapping of trends and hotspots in the field of exercise and cognition research over the past decade

**DOI:** 10.1007/s40520-023-02661-y

**Published:** 2024-02-03

**Authors:** Ying-Hai Zhu, Peng Hu, Ya-Xi Luo, Xiu-Qing Yao

**Affiliations:** 1 Department of Rehabilitation, The Second Affiliated Hospital of Chongqing Medical University, Chongqing, China; 2 Chongqing Municipality Clinical Research Center for Geriatric Medicine, Chongqing, China

**Keywords:** Bibliometric analysis, Physical exercise, Cognition

## Abstract

Exercise elicits a wide range of physiological responses in mammalian tissues that enhance a broad range of functions, particularly in improving cognitive performance. However, the field lacks a comprehensive bibliometric analysis that clarifies its knowledge structure and research hotspots. This study aims to address this gap and map the research landscape regarding the role of exercise in cognitive function enhancement. Firstly, the frequencies and co-occurrence of keywords were analysed to identify six main clusters: aging, cognitive impairment, rehabilitation, obesity, fatigue, and hippocampus. Secondly, reference timeline co-citation analysis revealed that hippocampus and aging were the major bursts with high intensity and long attention span while children had recently emerged as a topical subject. Finally, the evolution of themes from 2012 to 2022 was analysed, and found that older adults had been the leading research theme for exercise affecting cognition. Childhood obesity was an emerging theme that attracted increasing research attention in recent years while the hippocampus research theme expanded rapidly during the decade but remained a niche topic with less relevance to others. This research identified and summarised research priorities and evolutionary trends in exercise to improve cognition by constructing knowledge networks through visual analysis. It provides researchers with a comprehensive insight into the current state of the field to facilitate further research.

**Supplementary Information:**

The online version contains supplementary material available at 10.1007/s40520-023-02661-y.

## Introduction

Cognitive function is an essential human physiological function that encompasses memory, language, visual-spatial abilities, executive functions, computational skills, and comprehension judgments [1]. Cognitive impairment may occur at the full range of ages for different reasons at different stages of life. In children, the reasons for cognitive impairment include premature birth [2], adverse childhood experiences [3], etc. Obesity was also an essential factor for cognitive impairment in children. With the continuous progression of economic and social development, obesity-induced cognitive impairment in children has attracted increased attention from the public [4]. Among older adults, Alzheimer’s disease is the most common subtype of dementia which causes cognitive impairment in patients [5]. According to the latest public statement from the World Health Organization, more than 55 million people worldwide suffer from dementia and there are nearly 10 million new cases each year. The global economic cost of dementia was 1.3 trillion dollars in 2019 and about half of that cost was related to the care provided by informal family caregivers, who spent an average of five hours involved in care and supervision daily, placing a huge financial and care burden on families and society [6].

Exercise can trigger a profound physiological response in many organs, and it is widely accepted that it can enhance human health [7, 8]. Recent studies have focused on the impact of exercise on cognitive function and found that exercise was particularly beneficial for cognitive functions that rely on the hippocampus [9]. The main types of exercise are aerobic and resistance exercise, with a few researchers exploring specialised exercises like Tai chi and Qigong [10]. Aerobic exercise has the advantage of improving cardiovascular health by increasing the maximum oxygen consumption and increasing cardiovascular fitness while resistance exercise focuses on muscle mass and strength. Interestingly, both aerobic and resistance exercises have a beneficial impact on the improvement of cognitive function [11, 12]. The modality of exercise affects specific types of cognition with resistance exercise having a significant interactive effect on executive function, memory, and attention [13], while aerobic exercise mainly improves working memory [14].

In the past decade, over 30,000 publications have explored the correlation between exercise and cognition [15]. However, the kinds of literature are highly heterogeneous and cover a wide range of areas. To better understand the current hotspots and interconnections across the field, scholars have developed a “research weaving” that uses bibliometrics and knowledge mapping for visual analysis [16]. This approach allows quantitative analysis of large-scale, highly heterogeneous literature and provides an objective, visual display of past academic research output, thus reducing bias in evaluating scientific publications and helping researchers accurately capture research trends and hot topics in the field. It has been applied to various fields, including mental diseases [17], cardiovascular diseases [18] and degenerative diseases [19].

An increased emphasis has been placed on the study of cognitive enhancement through physical activity, with a dramatic increase in the relevant literature over the last decade. An objective quantitative study of the current status and trends of exercise on cognition is lacking with respect to large-scale scientific findings. Thus, the bibliometric analysis was conducted using data from the Web of Science Core Collection (WoSCC) to visualise the current state and trends of research in this area. With the analysis of the literature from 2012 to 2022, the current status of research in the field was summarised, hotspots and themes were uncovered, and future trends were predicted, ultimately addressing research gaps and plotting research landscapes on the role of exercise in enhancing cognitive functions.

## Methods

### Searching strategy and data collection

Literature was searched on 28 March 2023 in the WoSCC database, one of the most impactful scientific databases with resources widely used for bibliometric analysis in the health/medical field [20]. The process of the literature search strategy was as follows: (1) the search terms listed in Supplemental Fig. 1 were used; (2) publication dates were from 1 January 2012 to 31 December 2022; literature from 2023 was excluded due to ongoing publications; (3) records available in WoSCC; (4) article or review type of publication, excluded other types of publications, included conferences, comment and editorials. All records were downloaded in “plain text” format to avoid continuous updating and changes. Data were collected on publications and included title, abstract, keywords, authors, institutions, countries and references.

### Data analysis and mapping

Citespace (6.1.R6), VOSViewer (1.6.19, Leiden University, Netherlands), and the bibliometrics package (3.2.1) in R (4.2.3, www.r-project.org/) were used for the analysis.

VOSViewer, based on a bibliometric analysis software developed by Van Eck and Waltman, extracts key information from numerous publications and is commonly used to construct co-authorship, co-citation and co-occurrence networks to visualise vast knowledge graphs of the literature [21]. The primary analyses in this study included co-authorship analysis of countries/regions, institutions, and authors as well as co-occurrence analysis of author keywords, and calculated the widely used quantitative indices of productivity, the H-index [22] and the G-index [23]. The co-authorship analysis was used to reflect the collaboration and contribution of countries/regions, institutions, and authors in the retrieved literature, and such collaboration is considered to exist when different authors, institutions, or countries/regions appear in a publication at the same time [24]. The thickness and length of the inter-nodal connections indicate the strength and correlation of the two-nodal connections. Co-occurrence analysis based on author keywords can identify densely populated keywords and detect research hotspots. To reduce the effect of synonyms on the results by merging them. In the visual map, the area of the nodes is positively proportional to the frequency of keyword occurrences, and the colour of the nodes is determined by the class of the nodes in the cluster analysis.

Reference co-citation analysis can discover the knowledge base of the field and its evolutionary process. CiteSpace is a bibliometric analysis and visualisation software developed by Professor Chao-Mei Chen [25]. In this study, CiteSpace presented the co-citation relationships of references and clusters in a timeline with parameters set to time slice (2012–2022), year per slice (1), node type (cited references), selection criteria (top *N* = 50) and pruning (Pathfinder). Modularity and Silhouette are two indexes used to evaluate the effectiveness of reference co-citation clustering. The network community structure obtained is indicated to be significant when *Q* > 0.3. The clustering results possess high reliability when the Silhouette is 0.7. Additionally, co-citation burst detection can reflect the evolution of the research topic over time [26].

The Bibliometrics package, by Aria M and Cuccurullo C, was developed to allow for a comprehensive scientific mapping analysis [27]. It was used in this research to analyse thematic evolution and thematic maps to reflect thematic evolution. Thematic evolution was represented by Sankey diagrams to indicate the evolution of a theme over different time slices, and thematic maps were constructed with the density index as the longitudinal coordinate and the centrality index as the latitude coordinate. Density indicates the strength of internal links between a keyword of a theme and centrality is the strength of links between a theme and other themes externally [28]. These maps were grouped into four quadrants: (I) Motor themes represent significant and well-developed themes; (II) Niche themes represent highly developed themes but are less connected to other themes; (III) Emerging or Declining themes represent possible emerging or declining themes with low internal and external connections; (IV) Basic themes are to be considered the foundation of the discipline and cross-cutting themes. Tracking between time slices was determined to identify emerging or declining trends in themes [27]. The software was also used to create a global distribution network of cognitive publications on the impact of physical activity [29].

All raw data used in the study were obtained from public databases and ethical approval was waived.

## Results

### Distribution of publications over time

According to our search strategy, 30,345 publications from 2012 to 2022 were eventually retrieved from the WoSCC, including 24,993 articles (82.4%) and 5352 reviews (17.6%). The number of publications increased annually in general, from 1411 in 2012 to 4355 in 2022, showing a growth of 208.6%.

### Hotspots of the keywords

**Fig. 1:**
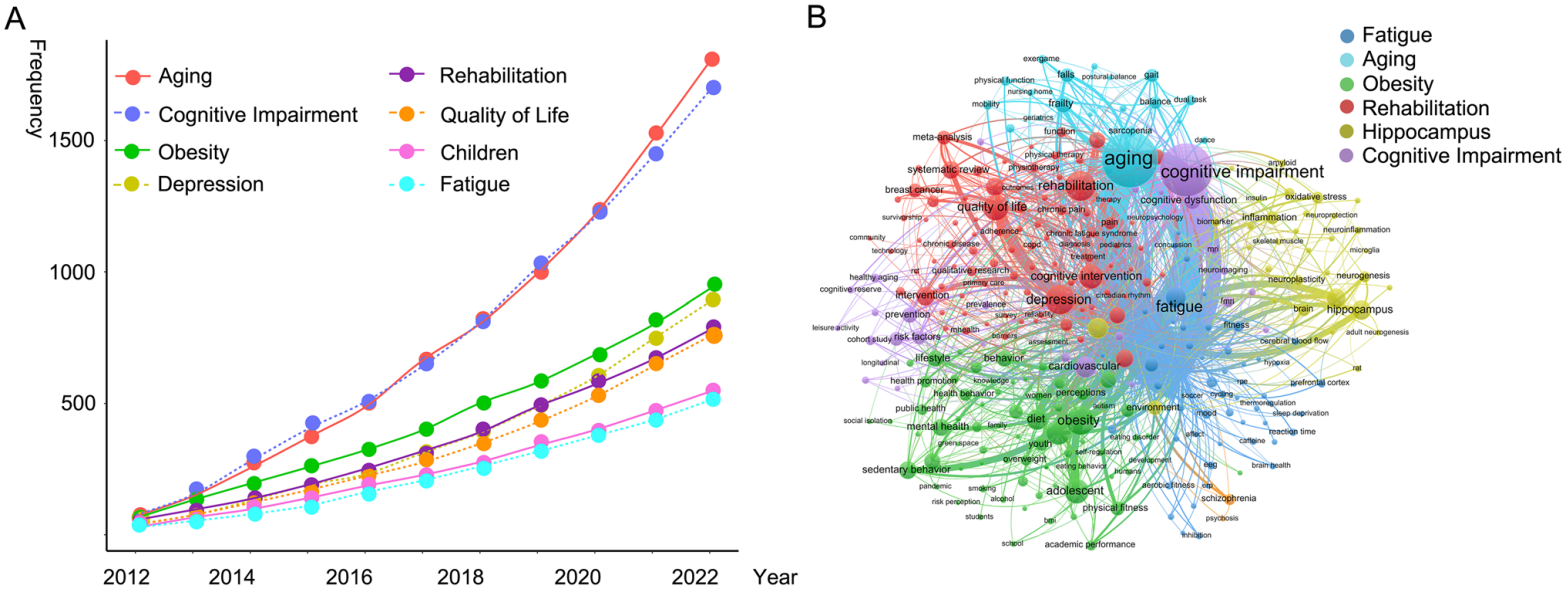
Trends and clusters of high-frequency keywords in the field of exercise and cognition research. (**a**) Changes of high-frequency keywords of publications in the field of exercise and cognition research over the last decades; (**b**) Keyword co-occurrence analysis of exercise and cognition, node size is proportional to the frequency of keyword occurrences, node colours are determined by the category of the node in the cluster analysis (colour figure online)

Initially, the keywords used in the literature in the last decade were analysed. In the keyword frequency statistics, due to the fact that exercise and cognition were inevitably the two most frequent keywords as search terms, high-frequency synonyms were removed from this analysis to avoid their impact on statistics and result interpretation. In the meanwhile, these synonyms were combined in the keyword clustering to make the visualisation more prominent and easier to interpret by eliminating clustering circles and text labels from both exercise and cognition search terms results presentation. Based on these data, changes in high-frequency keywords over time were calculated (Fig. 1a). Aging and cognitive impairment were found to be the two most frequent keywords associated with exercise and cognition searches, surpassing all other keywords significantly. Supplementary Table 1 listed the top 20 frequently occurring keywords. In addition to ‘exercise’ and ‘cognition’, rehabilitation, as well as sedentary behaviour, emerged as primary keywords.

The keyword co-occurrence analysis is shown in Fig. 1b. The selected keywords with the top 100 occurrences were clustered and grouped into six clusters. The blue cluster focused on fatigue-related studies with possible reasons including sleep deprivation, and dehydration, which could affect reaction time, mood and brain health. The green group was associated with obesity with factors related to sedentary behaviour like screen time, and lifestyle choices such as smoking or dieting/nutrition, being studied mainly in children and youth subjects. Interventions for weight loss in this context encompassed strategies like bariatric surgery and enhancing walking ability. The red cluster showed rehabilitation research focused on the quality of life, depression and cognitive interventions, with the main rehabilitation modality being physiotherapy and emerging technologies such as virtual reality were used to some extent in this area of research. Aging research was shown in the teal cluster where sarcopenia, frailty and falls were explored, all of which can be ameliorated through exercise and enhancement of gait balance. Finally, the yellow cluster highlighted hippocampus research focused on mechanisms involved in inflammation, neuroplasticity, oxidative stress, insulin, amyloid and tau.

### Trends of the keywords

**Fig. 2:**
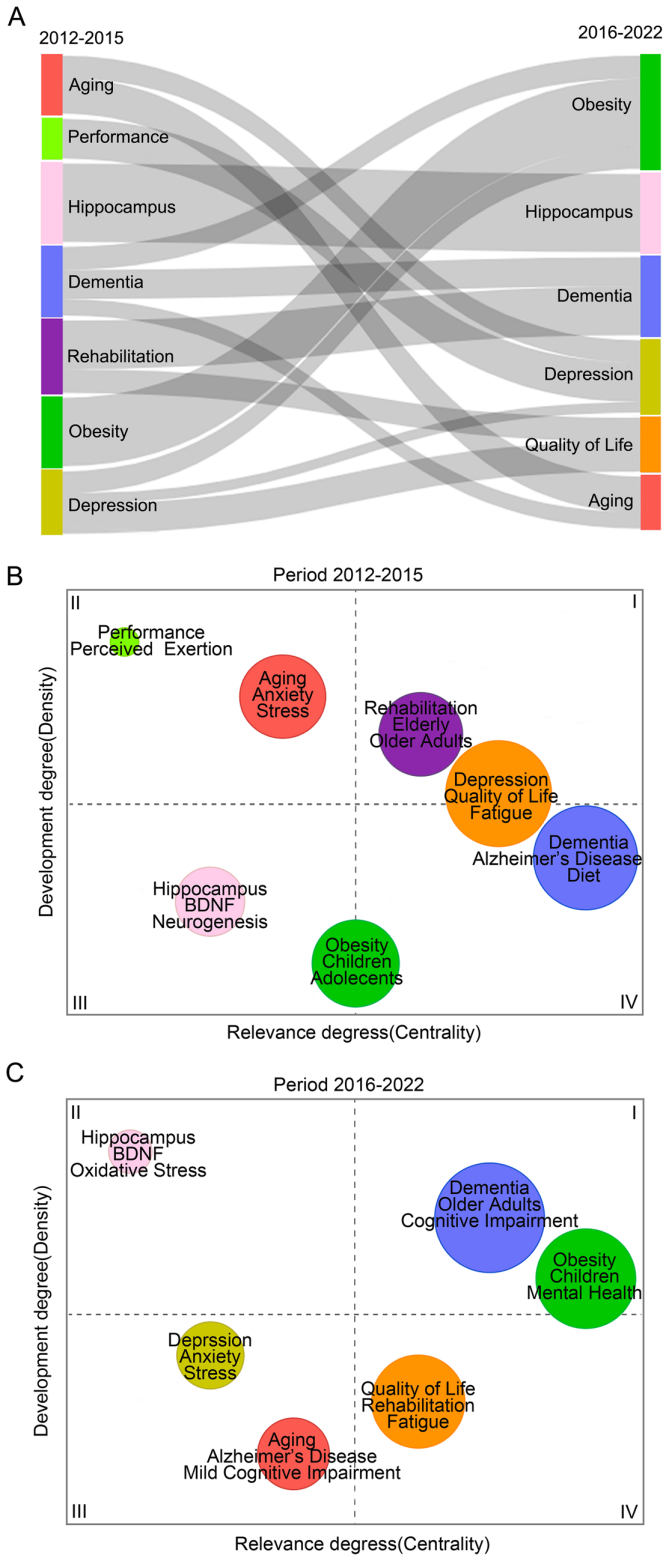
Thematic evolution and maps in the field of exercise and cognition research. (**a**) The thematic evolution of publications in exercise and cognition research over the last decade; (**b**/**c**) Thematic maps across the 2012–2015 and 2016–2022 periods in the exercise and cognition research. Thematic maps were divided into four quadrants: **I** Motor Themes with high density and centrality. **II** Niche Themes with high density but low centrality. **III** Emerging or Declining Themes with low density and centrality. **IV** Basic Themes with low density but high centrality

Based on the results of the keyword frequency variation and the analysis of its clustering, the evolution analysis of keyword-related themes was conducted. Thematic evolution and maps were graphed to visualize trends in these themes between 2012–2015 and 2016–2022, using Sankey diagrams (Fig. 2a) and the maps based on centrality and density (Fig. 2b/c). The diversion of research thematic terms over time was visualised by Sankey diagrams. Between 2012 and 2015, there were seven thematic terms, which were aging, performance, hippocampus, dementia, rehabilitation, obesity, and depression. In contrast to the previous years, in 2016–2022, the emerging theme word was quality of life, aggregated by rehabilitation and depression, accompanied by the disappearance of the theme words rehabilitation and performance, with all of the research on the performance theme word merging into the depression theme. Apart from depression, dementia had the highest number of shunts to obesity, dementia, and aging. The hippocampus remained unchanged across two time periods. Thematic maps present a more specific and clear view of thematic centrality and density variations. The finding indicated that the obesity and children theme became more prominent over time, moving from a low-density quadrant to a more centrality and density motor theme. The older adult group remained to be an important research theme with increasing centrality and density over the decade. Dementia and cognitive impairment have also emerged as vital themes related to aging populations, both of which were developed from the previous basic theme. Due to the high centrality and density of the three themes in this node, researchers could gain insights into the direction of their research themes from it. Interestingly, hippocampus and brain-derived neurotrophic factor (BDNF) started to emerge in this research field during 2011–2015, but the lack of centrality and density suggested that it had not yet received widespread attention, after seven years of development, both themes increased in density to the highest level but remained niche themes with less relevance to other themes.

### Bursts of the cited references

**Fig. 3:**
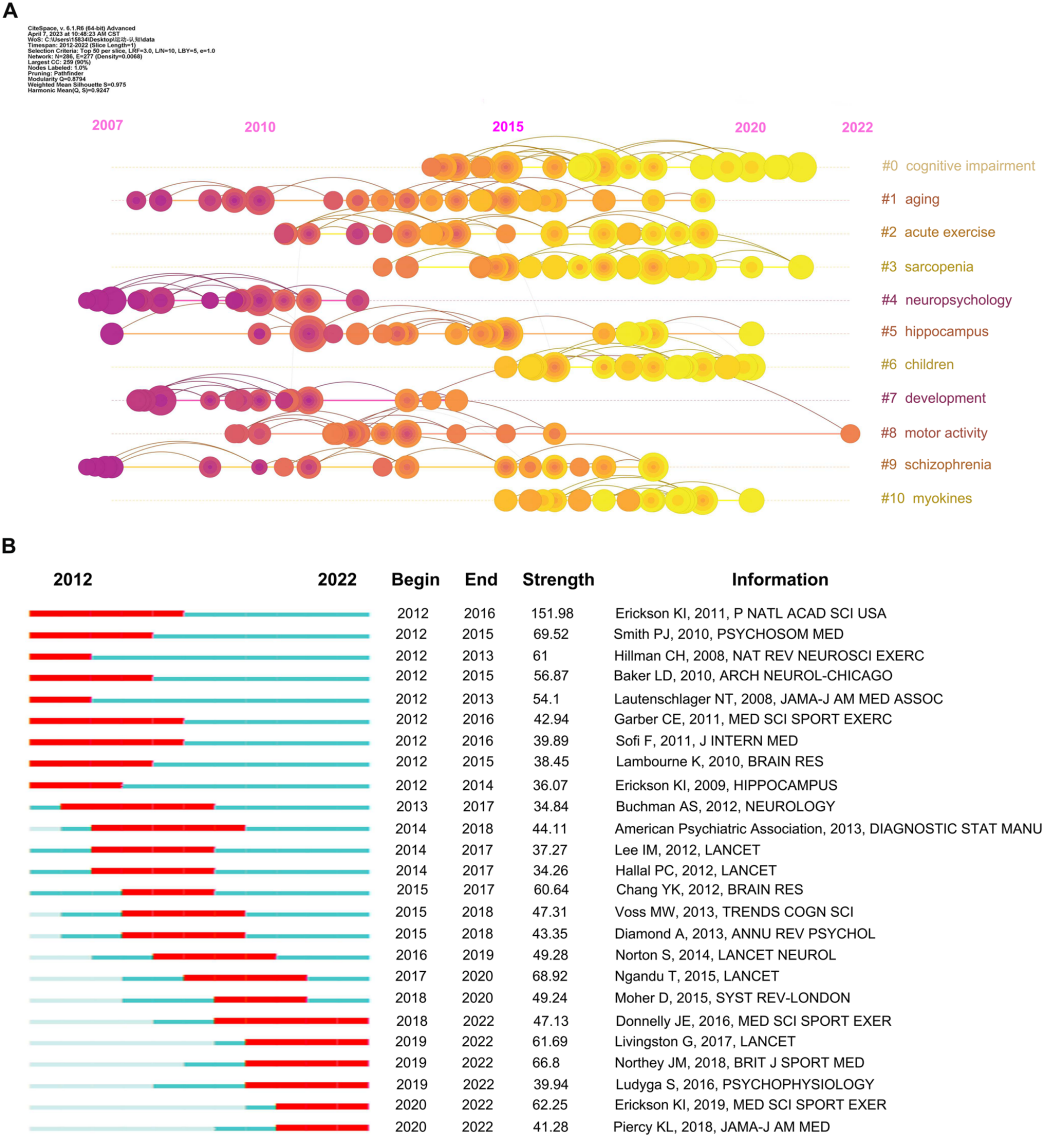
The burst of cited references in the field of exercise and cognition research. (**a**) The timeline co-citation analysis of references in exercise and cognition research, with node size proportional to the number of co-references in the literature and links between the literature, is expressed as link density. (**b**) Top 25 references with strong citation bursts in exercise and cognition reference, with red bars indicating the time interval between the start and end of reference co-citation bursts (colour figure online)

References to publications are the original repository for obtaining a knowledge network map of publications, and a reference co-citation analysis was conducted and presented as a timeline. Figure 3a shows the eleven main clusters from the reference co-citation analysis, with *Q* = 0.8794, indicating a significant cluster structure, and *S* = 0.975, reflecting a high level of reliability in the clustering outcomes. Significant clustering in co-citation links for hippocampus references occurred in 2011, 2015, and 2018, indicating important changes in foundational knowledge. Children emerged as the most recent reference co-citation cluster starting from 2015 until 2020. Apart from the hippocampus and children, aging was another long-lasting cluster with a significant amount of co-citation.

Co-citation bursts reflected the time interval between rapid changes in the intensity of references being cited, and the top 25 references with strong citation bursts were ranked according to the onset of the burst (Fig. 3b, Supplementary Table 2). The major types of publications were clinical studies (6) and reviews (12). The reference that ranked first in terms of cited burst intensity was “Exercise training increases the size of the hippocampus and improves memory” by Kirk I. Erickson’s team. This publication demonstrated that aerobic exercise training effectively reversed hippocampal volume loss in older adults and modulated memory function, providing solid evidence that exercise does improve cognitive function [30]. Among the top 25 references with strong citation burst, 6 remain widely cited until 2022. These references were mainly related to academic performance in children, dementia management, brain outcomes and exercise guidelines.

### Attribution and collaboration of countries/regions

A total of 158 countries/regions were actively contributing to the field. Information on publications in this area was compiled and analysed by country/region (Supplementary Fig. 2A, Supplementary Table 3). The USA (8804 publications) had emerged as the most productive country, while Australia led with the highest number of publications per million people and per trillion gross domestic product(GDP), at 104.83 and 1734.43 respectively. Co-authorship between countries/regions was depicted in Supplementary Fig. 2B, where five coloured clusters represented collaboration patterns, with link thickness indicating total link strength (TLS). The USA topped the list with a TLS of 5548, followed by England (TLS = 4679) and Australia (TLS = 3141). The red cluster revolved around the USA, Canada dominated the blue cluster, while three other clusters comprised mainly European countries centred on England, Spain and Germany. The visual map presented in Supplementary Fig. 2G provided an intuitive representation of cooperation between countries/regions.

## Attribution and collaboration of authors and institutions

Details about the top 10 authors with the most publications were collated in Table 1. Supplementary Fig. 2C displayed co-authorship between scholars, which was classified into 39 clusters. Charles H. Hillman had the highest number of articles, as well as the highest H-index and G-index, and TLS score. Meanwhile, Davy Vancampfort was the author with the highest average number of citations. Supplementary Table 4 listed the top 10 most productive institutions in this research area. The University of Illinois held the highest number of publications and the University of British Columbia had the highest citation rate and average citation per publication. Supplementary Fig. 2D displayed co-authorship relationships among these institutions, which had been grouped into nine clusters. Karolinska Institutet achieved the highest TLS (TLS = 1382) among all institutions.

**Table 1:** The top 10 productive authors in the field of exercise and cognitive function

Rank	Author	Affiliation	Numbers of publications	Numbers of citations	Average citations per publication	Co-authorship total link strength	H-index	G-index
1	Charles H Hillman	University of Illinois	108	5228	48.41	435	37	71
2	Arthur F Kramer	University of Illinois	99	5006	50.57	386	35	70
3	Robert W Motl	University of Illinois	92	2289	24.88	114	29	49
4	Teresa Liu-Ambrose	University of British Columbia	83	2595	31.27	239	27	49
5	Y K Chang	University of North Carolina at Greensboro	75	2697	35.96	195	32	60
6	Paul D Loprinzi	University of Mississippi	72	1414	19.64	57	27	49
7	Kirk I. Erickson	University of Pittsburgh	70	3800	54.29	167	35	66
8	Brendon Stubbs	King’s College London	66	3338	50.58	225	31	59
9	Davy Vancampfort	Katholieke Universiteit Leuven	60	3306	55.10	211	30	59
10	Hiroyuki Shimada	National Center for Geriatrics and Gerontology (Japan)	60	1539	25.65	267	22	38

### Distribution of disciplines and journals

The top 10 disciplines in publications were identified based on WoSCC categories, as shown in Supplementary Fig. 2E, Supplementary Table 5. The three most prominent disciplines were neuroscience, sports science and public environmental occupational health, accounting for approximately 36.49% of the total publications.

Supplementary Fig. 2F and Supplementary Table 6 illustrated the cumulative pattern of growth in annual publications and information on the top 10 most productive journals. The International Journal of Environmental Studies and Public Health published the highest number of papers (1130). The further analysis shown in Table 2 revealed that the Mini-Mental State Scale designed by MF Folstein et al. and published in J Psychiatr Res in 1975 was cited most frequently due to its rapid detection, accessibility, and acceptability [31]. Meanwhile, the highest average annual citation was Kirk I Erickson et al. who published a randomised controlled trial (RCT) in Proc Natl Acad Sci USA in 2011 and reported the effects of exercise on improving memory [30].

**Table 2:** Top 10 publications with high citations in the field of exercise and cognitive function

Rank	Publication	Source	Year	Total citations	Average citations per year	Doi
1	“Mini-mental state”. A practical method for grading the cognitive state of patients for the clinician	J Psychiatr Res	1975	1495	31.8	10.1016/0022-3956(75)90026-6
2	Exercise training increases size of hippocampus and improves memory	Proc Natl Acad Sci USA	2011	1369	124.4	10.1073/pnas.1015950108
3	Fitness effects on the cognitive function of older adults: a meta-analytic study	Psychol Sci	2003	1118	58.8	10.1111/1467-9280.t01-1-01430
4	Statistical Power Analysis for the Behavioral Sciences	N.A	1988	1037	30.5	10.4324/9780203771587
5	International physical activity questionnaire: 12-country reliability and validity	Med Sci Sports Exerc	2003	837	44.0	10.1249/01.MSS.0000078923.96621.1D
6	Be smart, exercise your heart: exercise effects on brain and cognition	Nat Rev Neurosci	2008	811	57.9	10.1038/nrn2298
7	Psychophysical bases of perceived exertion	Med Sci Sports Exerc	1982	785	19.6	10.1249/00005768-198205000-00012
8	Caring for older Americans: the future of geriatric medicine	J Am Geriatr Soc	2005	655	38.5	10.1111/j.1532-5415.2005.53350.x
9	Associations of depression with C-reactive protein, IL-1, and IL-6: a meta-analysis	Psychosom Med	2009	645	49.6	10.1097/PSY.0b013e3181907c1b
10	Phenotype of frailty: characterization in the women’s health and aging studies	J Gerontol A Biol Sci Med Sci	2006	630	39.3	10.1093/gerona/61.3.262

## Discussion

In this study, a knowledge mapping and bibliometric analysis of research on the effects of exercise on cognition from 2012 to 2022 was conducted. The number of global publications in this area has increased rapidly over the past decade. In our study, a large number of highly cited or co-cited publications focused on and summarised the role of physical activity in improving cognitive function, indicating this was a long-standing and widespread concern. The hippocampus and aging were the hot spots in the field with high intensity and duration. Additionally, children have become an increasingly important topic in recent years.

The hippocampus is a crucial structure that regulates various cognitive functions, such as memory, learning, and spatial navigation, which are influenced by adult neurogenesis [32], long-term potentiation [33] and pattern separation [34]. A wealth of research suggests that exercise significantly influences hippocampal structure and function. In an RCT that included 86 women aged 70–80 years, aerobic training was shown to significantly increased hippocampal volume in older women with suspected mild cognitive impairment, and increased hippocampal volume was independently associated with reduced verbal memory and learning performance [35]. In recent years, increasing numbers of researchers have delved into this hotspot and found that the mechanisms included: secretion of growth factors such as BDNF [36, 37], neuroplasticity enhancement [38], improved brain oxygenation and nutrient supply [39], and reduced inflammation to protect neurons [40]. Recent research has shown that different forms of exercise affect hippocampal function differently. For example, aerobic exercise improved hippocampal volume and spatial memory [41, 42], while resistance exercise enhanced hippocampal neuroplasticity and learning ability [43]. Further research is required to determine the optimal type of exercise or combination of them for achieving maximum benefits [44]. Other factors such as duration, intensity, frequency [45], gender [46], age, and baseline cognitive function [35] also influenced the effects of exercise on the hippocampus’s function. Therefore, more research should be conducted to identify personalised exercise intervention programs that are applicable to different populations to maximise hippocampal function improvement [47, 48].

Aging was another topic identified in this study that received a long attention span and a high co-citation rate. Aging processes were often accompanied by an increase in frailty [49], falls [50], and sarcopenia [51], which were risk factors for head injury or stroke and can reduce the ability of older people to participate in physical activity and may lead to a decline in cognitive function [52–54]. Nevertheless, exercise had been shown to positively impact the aging process by combating typical signs such as frailty, balance issues, and sarcopenia [55]. Exercise, especially multi-component exercise, can prevent, delay and reverse frailty, and improve the physical fitness of pre-frail/frail older adults [56, 57]. Regular physical activity improved physical function and balanced performance, reducing the risk of falls among older individuals [58, 59]. Proper strength training can help combat sarcopenia by preventing muscle loss and increasing both muscle mass and strength [60]. Additionally, regular exercise has been shown to improve cognitive function in older people [61]. In conclusion, frailty, falls and muscle loss during aging may negatively affect cognitive function; however, exercise can mitigate these effects and improve cognitive function. Therefore, it is recommended that older adults engage in regular exercise to improve frailty, reduce falls and muscle loss, enhance physical and cognitive function, and ultimately achieve the goal of delaying aging [8, 14].

Children are in a crucial stage of cognitive development, various drugs [62], innate diseases [63], and obesity [4] may affect their cognitive development and cause cognitive disorders. Childhood obesity is an emerging research hotspot that has become a major public health issue with increasing prevalence worldwide, capable of causing cognitive impairment and mental health problems in children [64, 65]. Studies have shown that obese children tended to have poorer cognitive function than their normal-weight peers, particularly in memory, attention and learning areas [66]. This may be due to chronic inflammation, oxidative stress and insulin resistance caused by obesity [67]. Regular exercise had been shown to have a positive impact on cognitive function in children by increasing blood flow and oxygen supply to the brain. In particular, modified executive function by aerobic exercise played an essential role in complex behaviour with the potential to contribute to successful academic and occupational achievement as well as social interaction [68]. A study of 250 Spanish schoolchildren found that their academic performance was associated with their physical activity [69]. Moreover, exercise also improves mental health in children, including reduced depressive symptoms [70]. Encouraging regular physical activity and promoting healthy lifestyles may have significant benefits for both physical and cognitive health in children. In summary, physical activity had a significant impact on obese children’s cognitive development and academic performance. Further research is needed to explore the underlying mechanisms of this relationship and to develop effective interventions that promote their physical health, improve cognitive function in obese children, and facilitate their academic performance.

In addition to the keywords discussed above, rehabilitation and fatigue were the main clusters for the keywords in this review. Fatigue was reported as a prevalent complaint in older adults and was associated with their negative functional outcomes [71], with perceived fatigue being more severe in patients with mild cognitive impairment than in normal individuals [72]. Whether it is possible to improve fatigue, and hence cognitive performance, through exercise is lacking in high-quality article reports, and more researches are needed to explore this. Rehabilitation is an important approach to cognitive intervention and improving quality of life, with exercise being one of many rehabilitation treatments [73]. In the future, using various rehabilitation tools, including exercise, to improve the quality of life for people with cognitive impairment is one of the concerns that deserves the attention of researchers.

Finally, the contributions and collaborations of countries/regions, authors and institutions were analysed, as well as the distribution of publications across disciplines and journals. In the last decade, the USA, England and China have been the main producing countries/regions in this field. Additionally, the institutions with the largest number of publications and the highest quality of literature were mainly located in Europe and the United States. The reasons for this distribution were closely related to the aging process, with the elderly being a high-incidence cohort of cognitive impairment [74]. Only with the dedication of more researchers can this therapeutic challenge be solved at an early date. Research related to exercise and cognition was a multidisciplinary field. Our results found that the field was mainly focused on neuroscience, sport science and public environmental occupational health, accounting for 36.49% of the literature in the field.

A limitation of this study is that the WoSCC database was the only data source for bibliometric searches. Although WoSCC is currently the primary data source for bibliometric analysis, the retrieved data may be incomplete due to its consistency and standardised format of bibliographic records and broad coverage [75]. The current difficulty in achieving effective integration of indexed records of large-scale publications across databases remains unresolved in bibliometric analysis studies [76, 77]. Suitable tools are needed in the future to address this limitation to reduce selection bias.

## Conclusion

In conclusion, our comprehensive review of 30,345 publications in the field of exercise and cognition highlights the emerging research areas of the hippocampus and aging, along with the growing focus on studying children. Multiple studies have consistently demonstrated the beneficial effects of exercise on hippocampal function and cognitive abilities. However, despite these promising findings, many cognitive disorders still pose significant challenges. Therefore, future research should prioritize the investigation of the precise mechanisms underlying cognitive enhancement through exercise, aiming to optimize cognitive performance.

### Supplementary Information

Below is the link to the electronic supplementary material.

Supplementary file1 (DOCX 1749 KB)

## Data Availability

All raw data was obtained from the Web of Science Core Collection. Data are available upon reasonable request.
