# Supplementary material for: Knowledge mapping of trends and hotspots in the field of exercise and cognition research over the past decade
Source: Aging Clin Exp Res. 2024 Feb 3;36(1):19. doi: 10.1007/s40520-023-02661-y (PMC10838253; doi:10.1007/s40520-023-02661-y)
Supplement: Supplementary file 1 — Supplementary file1 (DOCX 1749 KB) [file 40520_2023_2661_MOESM1_ESM.docx]

**Article Title** Knowledge Mapping of Trends and Hotspots in the Field of Exercise and Cognition Research Over the Past Decade

**Author Names** Ying-Hai Zhu ^1^, Peng Hu^1^, Ya-Xi Luo^1^, Xiu-Qing Yao^1,2^

**Address Correspondence to**

Xiu-Qing Yao, Department of Rehabilitation, The Second Affiliated Hospital of Chongqing Medical University, Chongqing, China. Tel: +86 (23) 63693883. Email: [dryaoxq@cqmu.edu.cn](mailto:dryaoxq@cqmu.edu.cn). ORCID: 0000-0002-0292-2792

Ya-Xi Luo, Department of Rehabilitation, The Second Affiliated Hospital of Chongqing Medical University, Chongqing, China. Tel: +86 (0) 15683686823. Email: luoyaxi@hospital.cqmu.edu.cn. ORCID: 0000-0001-7198-8720

**Supplemental Figure 1** The flowchart of publication searching and screening

**
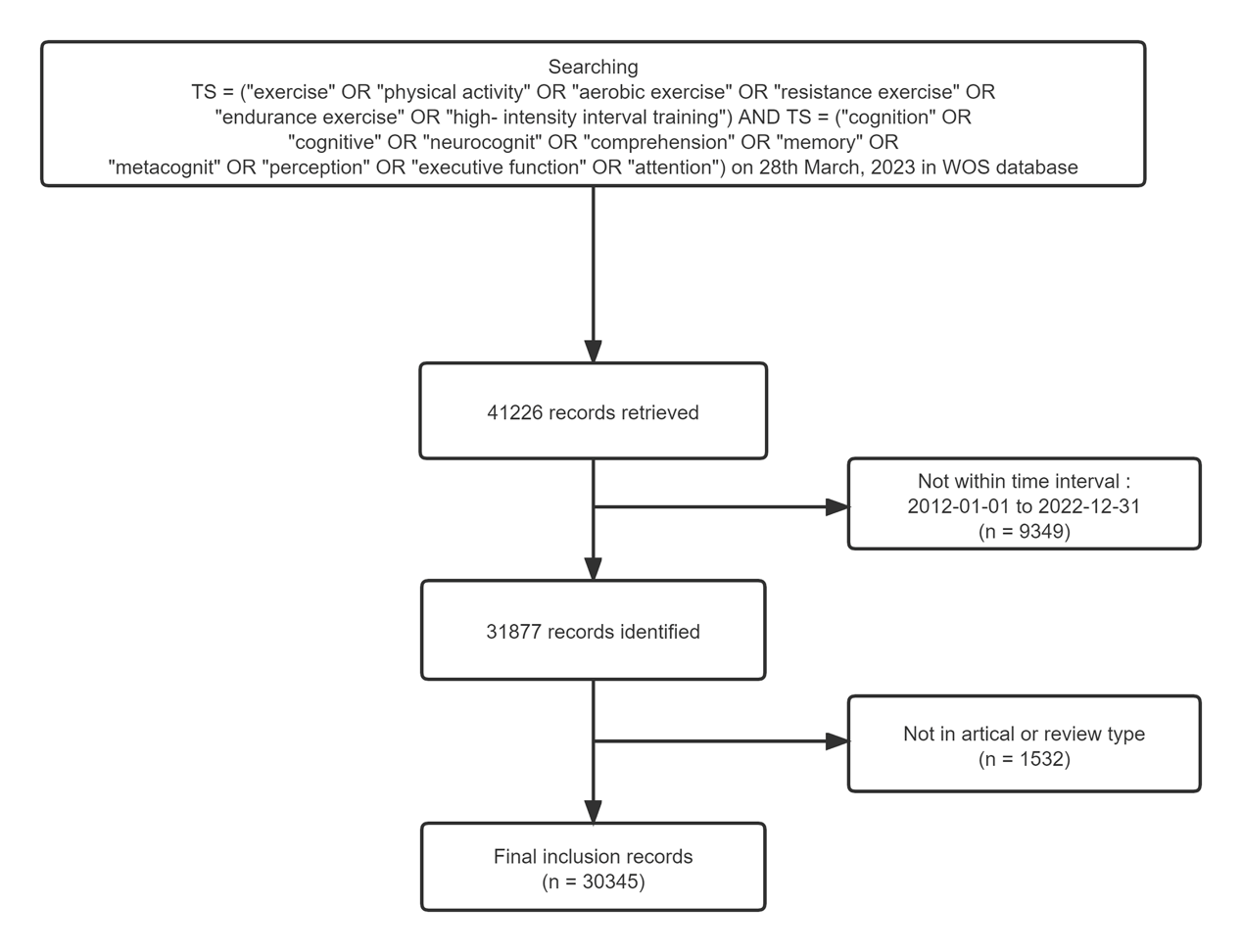
**

**Supplemental Table 1** Top 20 authors’ keywords in the field of exercise and cognition research

| **Rank** | **Keywords** | **Records** | **Total link strength** | **Rank** | **Keywords** | **Records** | **Total link strength** |
| --- | --- | --- | --- | --- | --- | --- | --- |
| 1 | exercise | 9885 | 22656 | 11 | adolescent | 667 | 1715 |
| 2 | cognition | 4495 | 11278 | 12 | cardiovascular | 533 | 1409 |
| 3 | aging | 2792 | 7014 | 13 | diet | 516 | 1614 |
| 4 | cognitive impairment | 2532 | 6203 | 14 | diabetes | 506 | 1275 |
| 5 | rehabilitation | 995 | 2481 | 15 | fatigue | 498 | 1290 |
| 6 | depression | 956 | 2834 | 16 | hippocampus | 448 | 1278 |
| 7 | obesity | 904 | 2460 | 17 | bdnf | 445 | 1195 |
| 8 | quality of life | 746 | 1929 | 18 | randomized controlled trial | 427 | 1292 |
| 9 | child | 703 | 1868 | 19 | intervention | 413 | 1255 |
| 10 | cognitive intervention | 682 | 1862 | 20 | sedentary behavior | 398 | 1165 |

**Supplemental Table 2** Top 25 references with strong citation burst in the field of exercise and cognition research

| **References** | **Year** | **Strength** | **Begin** | **End** | **Type** | **Journal** | **DOI** |
| --- | --- | --- | --- | --- | --- | --- | --- |
| Exercise training increases size of hippocampus and improves memory | 2011 | 151.98 | 2012 | 2016 | RCT | Proc Natl Acad Sci U S A | 10.1073/pnas.1015950108 |
| Aerobic exercise and neurocognitive performance: a meta-analytic review of randomized controlled trials | 2010 | 69.52 | 2012 | 2015 | Review | Psychosom Med | 10.1097/PSY.0b013e3181d14633 |
| Be smart, exercise your heart: exercise effects on brain and cognition | 2008 | 61 | 2012 | 2013 | Review | Nat Rev Neurosci | 10.1038/nrn2298 |
| Insulin resistance and Alzheimer-like reductions in regional cerebral glucose metabolism for cognitively normal adults with prediabetes or early type 2 diabetes | 2010 | 56.87 | 2012 | 2015 | RCT | Arch Neurol | 10.1001/archneurol.2010.225 |
| Effect of physical activity on cognitive function in older adults at risk for Alzheimer disease: a randomized trial | 2008 | 54.1 | 2012 | 2013 | RCT | JAMA | 10.1001/jama.300.9.1027 |
| American College of Sports Medicine position stand. Quantity and quality of exercise for developing and maintaining cardiorespiratory, musculoskeletal, and neuromotor fitness in apparently healthy adults: guidance for prescribing exercise | 2011 | 42.94 | 2012 | 2016 | Practice Guideline | Med Sci Sports Exerc | 10.1249/MSS.0b013e318213fefb |
| Physical activity and risk of cognitive decline: a meta-analysis of prospective studies | 2011 | 39.89 | 2012 | 2016 | Review | J Intern Med | 10.1111/j.1365-2796.2010. 02281.x |
| Comparison between proliferative and neuron-like SH-SY5Y cells as an in vitro model for Parkinson disease studies | 2010 | 38.45 | 2012 | 2015 | Comparative study | Brain Res | 10.1016/j.brainres.2010.03.102 |
| Aerobic fitness is associated with hippocampal volume in elderly humans | 2009 | 36.07 | 2012 | 2014 | Article | Hippocampus | 10.1002/hipo.20547 |
| Total daily physical activity and the risk of AD and cognitive decline in older adults | 2012 | 34.84 | 2013 | 2017 | RCT | Neurology | 10.1212/WNL.0b013e3182535d35 |
| Diagnostic and statistical manual of mental disorders: DSM-5™, 5th ed. | 2013 | 44.11 | 2014 | 2018 | Guideline | Diagnostic Stat Manu | 10.1176/appi.books.9780890425596 |
| The pandemic of physical inactivity: global action for public health | 2012 | 37.27 | 2014 | 2017 | Review | Lancet | 10.1016/S0140-6736(12)60898-8 |
| Global physical activity levels: surveillance progress, pitfalls, and prospects | 2012 | 34.26 | 2014 | 2017 | Review | Lancet | 10.1016/S0140-6736(12)60646-1 |
| The effects of acute exercise on cognitive performance: a meta-analysis | 2012 | 60.64 | 2015 | 2017 | Meta-Analysis | Brain Res | 10.1016/j.brainres.2012.02.068 |
| Bridging animal and human models of exercise-induced brain plasticity | 2013 | 47.31 | 2015 | 2018 | Review | Trends Cogn Sci | 10.1016/j.tics.2013.08.001 |
| Executive functions | 2013 | 43.35 | 2015 | 2018 | Review | Annu Rev Psychol | 10.1146/annurev-psych-113011-143750 |
| Potential for primary prevention of Alzheimer's disease: an analysis of population-based data | 2014 | 49.28 | 2016 | 2019 | Meta-Analysis | Lancet Neurol | 10.1016/S1474-4422(14)70136-X |
| A 2 year multidomain intervention of diet, exercise, cognitive training, and vascular risk monitoring versus control to prevent cognitive decline in at-risk elderly people (FINGER): a randomised controlled trial | 2015 | 68.92 | 2017 | 2020 | RCT | Lancet | 10.1016/S0140-6736(15)60461-5 |
| Preferred reporting items for systematic review and meta-analysis protocols (PRISMA-P) 2015 statement | 2015 | 49.24 | 2018 | 2020 | Article | Syst Rev | 10.1186/2046-4053-4-1 |
| Physical Activity, Fitness, Cognitive Function, and Academic Achievement in Children: A Systematic Review | 2016 | 47.13 | 2018 | 2022 | Review | Med Sci Sports Exerc | 10.1249/MSS.0000000000000901 |
| Exercise interventions for cognitive function in adults older than 50: a systematic review with meta-analysis | 2018 | 66.8 | 2019 | 2022 | Review | Br J Sports Med | 10.1136/bjsports-2016-096587 |
| Dementia prevention, intervention, and care | 2017 | 61.69 | 2019 | 2022 | Review | Lancet | 10.1016/S0140-6736(17)31363-6 |
| Acute effects of moderate aerobic exercise on specific aspects of executive function in different age and fitness groups: A meta-analysis | 2016 | 39.94 | 2019 | 2022 | Review | Psychophysiology | 10.1111/psyp.12736 |
| Physical Activity, Cognition, and Brain Outcomes: A Review of the 2018 Physical Activity Guidelines | 2019 | 62.25 | 2020 | 2022 | Review | Med Sci Sports Exerc | 10.1249/MSS.0000000000001936 |
| The Physical Activity Guidelines for Americans | 2018 | 41.28 | 2020 | 2022 | Guideline | JAMA | 10.1001/jama.2018.14854 |

**
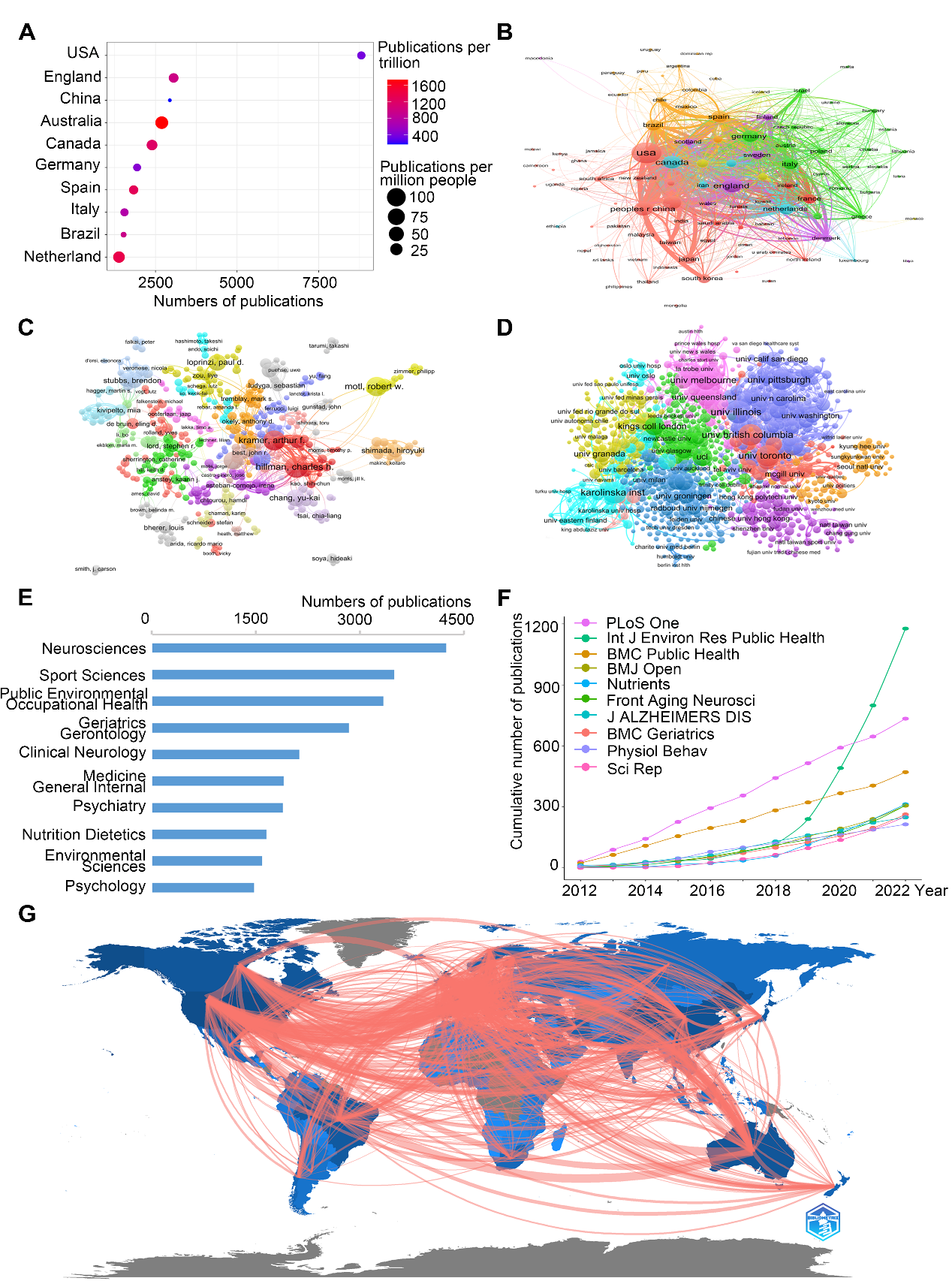
**

**Supplemental Figure 2** Attribution sources, collaborative networks and publication distribution in the field of exercise and cognition research

(A) Top 10 most productive countries/regions in the field of exercise and cognition research; (B) Co-authorship relationships among countries/regions, with the size of nodes indicating the number of publications made, and the thickness and length of links between nodes indicating the strength and relevance of links between nodes; (C) Co-authorship relationships among authors; (D) Co-authorship relationships among institutions; (E) Distribution of publications by discipline; (F) Top 10 prolific journals in terms of the cumulative growth patterns; (G) Map visualization of countries/regions co-authorship relationships in the field of exercise and cognition research, with the thickness of links between nodes indicating the degree of cooperation between countries, with thicker signifying closer

**Supplemental Table 3** The top 10 productive countries/regions in the field of exercise and cognition research

| **Rank** | **Country/**  **region** | **Numbers of**  **Publications** | **Publications**  **per million people*** | **Publications**  **per trillion GDP*** | **Numbers of**  **citations** | **Average citations per publication** | **Co-authorship**  **total link strength** |
| --- | --- | --- | --- | --- | --- | --- | --- |
| 1 | USA | 8804 | 26.53 | 377.61 | 251340 | 28.55 | 5548 |
| 2 | England | 3053 | 45.35 | 974.97 | 98892 | 32.39 | 4679 |
| 3 | China | 2936 | 2.08 | 165.56 | 38194 | 13.01 | 1523 |
| 4 | Australia | 2693 | 104.83 | 1734.43 | 81879 | 30.4 | 3141 |
| 5 | Canada | 2392 | 62.54 | 1203.02 | 77710 | 32.49 | 2468 |
| 6 | Germany | 1937 | 23.28 | 454.70 | 51640 | 26.66 | 2702 |
| 7 | Spain | 1829 | 38.57 | 1281.37 | 38863 | 21.25 | 2477 |
| 8 | Italy | 1550 | 26.22 | 735.40 | 41595 | 26.84 | 2288 |
| 9 | Brazil | 1522 | 7.10 | 945.94 | 24883 | 16.35 | 1339 |
| 10 | Netherlands | 1381 | 78.77 | 1363.48 | 47210 | 34.19 | 2246 |

*Calculations based on 2021 population and GDP data from world bank (https://databank.worldbank.org/). GDP is calculated using GDP (current US$).

**Supplemental Table 4** The top 10 productive institutions in the field of exercise and cognition research

| **Rank** | **Institution** | **Country/**  **region** | **Numbers of**  **Publications** | **Numbers of**  **Citations** | **Average citations per publication** | **Co-authorship**  **total link strength** |
| --- | --- | --- | --- | --- | --- | --- |
| 1 | University of Illinois | USA | 432 | 17327 | 40.11 | 897 |
| 2 | Karolinska Institutet | Sweden | 403 | 12831 | 31.84 | 1382 |
| 3 | University of British Columbia | Canada | 397 | 20709 | 52.16 | 958 |
| 4 | University of Sydney | Australia | 396 | 19507 | 49.26 | 960 |
| 5 | [University of Toronto](https://www.utoronto.ca/) | Canada | 395 | 12240 | 30.99 | 1176 |
| 6 | University of Pittsburgh | USA | 345 | 12156 | 35.23 | 1020 |
| 7 | University of Melbourne | Australia | 332 | 10307 | 31.05 | 1127 |
| 8 | [Harvard Medical School](https://hms.harvard.edu/) | USA | 316 | 8299 | 26.26 | 1134 |
| 9 | University College London | England | 289 | 9360 | 32.39 | 976 |
| 10 | University of Sao Paulo | Brazil | 289 | 4392 | 15.20 | 421 |

**Supplemental Table 5** Top 20 categories in the field of exercise and cognition research

| **Rank** | **Record Count** | **Web of Science Categories** | **% Of 30,345** |
| --- | --- | --- | --- |
| 1 | 4243 | Neurosciences | 13.983 |
| 2 | 3493 | Sport Sciences | 11.511 |
| 3 | 3338 | Public Environmental Occupational Health | 11.000 |
| 4 | 2841 | Geriatrics Gerontology | 9.362 |
| 5 | 2126 | Clinical Neurology | 7.006 |
| 6 | 1903 | Medicine General Internal | 6.271 |
| 7 | 1891 | Psychiatry | 6.232 |
| 8 | 1650 | Nutrition Dietetics | 5.437 |
| 9 | 1588 | Environmental Sciences | 5.233 |
| 10 | 1472 | Psychology | 4.851 |
| 11 | 1456 | Rehabilitation | 4.798 |
| 12 | 1389 | Physiology | 4.577 |
| 13 | 1233 | Multidisciplinary Sciences | 4.063 |
| 14 | 1174 | Gerontology | 3.869 |
| 15 | 1061 | Health Care Sciences Services | 3.496 |
| 16 | 912 | Behavioral Sciences | 3.005 |
| 17 | 871 | Medicine Research Experimental | 2.87 |
| 18 | 813 | Nursing | 2.679 |
| 19 | 773 | Endocrinology Metabolism | 2.547 |
| 20 | 757 | Oncology | 2.495 |

**Supplemental Table 6** The top 10 productive journals in the field of exercise and cognition research

| **Rank** | **Source** | **Category** | **2021 impact factor** | **2021 JCR partition** | **Total publications** | **Numbers of**  **citations** | **Average citations per publication** | **H-index** | **G-index** |
| --- | --- | --- | --- | --- | --- | --- | --- | --- | --- |
| 1 | International Journal of Environmental Research and Public Health | Environmental Sciences/Public,Environmental & Occupational Health | 4.614 | Q3 | 1130 | 9025 | 7.99 | 40 | 61 |
| 2 | PLOS ONE | Multidisciplinary Sciences | 3.752 | Q3 | 712 | 16627 | 23.35 | 64 | 87 |
| 3 | BMC Public Health | Public health, environmental health & occupational health | 4.135 | Q3 | 453 | 10040 | 22.16 | 47 | 78 |
| 4 | Frontiers in Aging Neuroscience | Geriatrics&  Neurosciences | 5.702 | Q2 | 302 | 6023 | 19.94 | 42 | 67 |
| 5 | Nutrients | Nutrition& Dietetics | 6.706 | Q2 | 300 | 4395 | 14.65 | 37 | 54 |
| 6 | BMJ OPEN | Medicine,General & Internal | 3.006 | Q4 | 296 | 3026 | 10.22 | 27 | 43 |
| 7 | BMC Geriatric | geriatrics | 4.07 | Q1 | 256 | 3872 | 15.13 | 34 | 50 |
| 8 | Scientific Reports | Multidisciplinary Sciences | 4.996 | Q3 | 248 | 3456 | 13.94 | 31 | 47 |
| 9 | Journal of Alzheimers Disease | Neurosciences | 2.632 | Q3 | 247 | 6840 | 27.69 | 42 | 71 |
| 10 | Physiology & Behavior | Behavior science&Psychology: biology | 3.742 | Q3 | 204 | 3099 | 15.19 | 30 | 44 |
